# Beyond severity: utility as a criterion for setting the scope of RGCS

**DOI:** 10.1038/s41431-024-01640-9

**Published:** 2024-05-29

**Authors:** Lisa Dive, Anne-Marie Laberge, Lucinda Freeman, Eline M. Bunnik

**Affiliations:** 1 Graduate School of Health, University of Technology Sydney, Sydney, NSW Australia; 2 Department of Pediatrics, Université de Montréal, Montréal, QC Canada; 3 Department of Medical Ethics, Philosophy and History of Medicine, Erasmus MC, Rotterdam, The Netherlands

**Keywords:** Population genetics, Ethics

## Abstract

Reproductive genetic carrier screening (RGCS) allows prospective parents to identify and act upon their chances of having a child with a genetic condition. In deciding which genetic conditions to include in RGCS, severity is often used as a criterion. However, the concept is inherently complex, subjective and multidimensional, and determinations of severity will remain intractably contested. We propose the concept of utility as a criterion for setting the scope of RGCS, and put forward two central arguments for doing so. First, utility is a more appropriate and effective concept as it responds to context and makes an explicit connection between the purpose of RGCS and the value of information obtained for that purpose: namely, to facilitate reproductive decision-making. Utility comprises both clinical and personal utility, and varies according to the availability and accessibility of reproductive options, including pre-implantation genetic testing, prenatal genetic diagnosis, and termination of pregnancy. Second, there are ethical reasons for preferring utility over severity. Utility is a property of the information gleaned from RGCS, while severity is a property of a genetic condition or of an instance of this condition in a person. While consideration of the severity of genetic conditions is not lost when focusing on utility, the need to rely on value judgements regarding the quality of life of people who live with genetic conditions is circumvented. Therefore, utility should replace severity as justification for the inclusion of genetic conditions in RGCS programmes.

## Introduction

Genomic testing is now part of health care across all stages of the lifespan, from preconception through to end of life. Reproductive genetic carrier screening (RGCS) allows prospective parents to identify their chances of having children with a genetic condition, information that can be relevant for their reproductive decisions thereafter. There is international agreement about the benefit of offering RCGS to all women planning a pregnancy or in early pregnancy [1–4]. However, there is a lack of consensus on exactly which conditions should be included in the offer of carrier screening, and how that decision should be made [5]. Technology now allows RCGS to screen for hundreds of conditions concurrently. Panels of conditions may be tailored to people from specific ethnic backgrounds, but more commonly a pan-ethnic test is offered to anyone in the population [2]. RGCS is variously described as ‘expanded’ and/or ‘universal’ carrier screening, to signify that it screens for large numbers of genes and is offered widely in the population.

RGCS may be offered publicly through a health care system, or privately through commercial entities. Currently available offers of RGCS are most often on a user pays basis [6]. The commercial organisations have their own drivers influencing decisions about what to include in an offer of screening. In the absence of specific guidelines, companies often make decisions based on what is likely to make their screening offer more competitive in the marketplace. Governments in several jurisdictions, such as Australia and the Netherlands, are now exploring the possibility of offering publicly funded RGCS. The offer of screening through a government-funded programme requires careful consideration of what conditions to include to ensure responsible implementation. A public offer of screening has normative implications, as it suggests that the conditions included are worth taking steps to avoid. The decision is complex, particularly in a public health care system where there are many different stakeholders including health care providers, funders, and people who live with genetic conditions [7].

Mackenzie’s Mission was a government-funded pilot study that offered RGCS to ~10,000 Australian couples between 2018 and 2022, with the aim of exploring how to implement a population-wide offer of RGCS. Over 750 autosomal recessive and X-linked conditions were selected to include in Mackenzie’s Mission based on criteria that included: the condition should be life-limiting or disabling, with childhood onset, such that couples (“an ‘average’ couple”) would be likely to take steps to avoid having an affected child, and/or be one for which early diagnosis and intervention would substantially change the outcome [8]. These criteria acknowledge that severity is a central factor in deciding which conditions to include in RGCS, in addition to technical considerations such as penetrance and the strength of the genotype–phenotype association. It is widely agreed that severity is among the key inclusion criteria for deciding which conditions to include in an offer of RGCS [6]. Severity is also an inclusion criterion for other kinds of screening and testing in health care, so our analysis of severity is likely to have implications beyond the context of RGCS.

Using severity as an inclusion criterion for RGCS is an important part of addressing concerns about similarities with eugenics and for responding to the disability and expressivist critiques of population-wide RGCS [9]. Increasingly it is acknowledged that the most ethically acceptable aim for RGCS is to support reproductive autonomy by providing information relevant to decisions about reproduction [6]. However, there is also an impetus to avoid the suffering associated with some serious health conditions. Avoiding the birth of a child with a severe genetic condition is more ethically acceptable because it is clear that doing so avoids suffering – both for the child and for the family. While there is agreement about what constitutes a very severe condition or a very mild condition, there is a substantial ‘grey area’ in which it becomes difficult and complex to draw the line between those conditions that are severe and those that are mild [10]. Determining the severity of a specific condition (for purposes of deciding if it should be included in RGCS) is difficult because the concept of severity is internally complex and has many contributing factors. Furthermore, people’s experiences with genetic conditions – whether personal or professional – can vary and exert substantial influence on how they perceive the severity of particular conditions [11].

The criterion of severity needs to be considered both at the policy level and also in the context of the health decisions that prospective parents will make using information provided through RGCS. When policymakers are developing and designing the implementation of RGCS, they must rely on a generalised understanding of the severity of each genetic condition under consideration. By contrast, when prospective parents receive a finding from RGCS that is relevant to their health care decision-making, they require a richer understanding of the potential impact of that condition in the context of their specific family [10].

This paper presents two central arguments for using the concept of utility for setting the scope of RGCS, as a response to the operational challenges in relying on the concept of severity both at the policy level and in the context of patients’ decision-making. Our first argument is that utility encapsulates aspects of severity, but in a way that responds to the context of screening and the purpose of RGCS. Utility varies in proportion to the severity of the condition in question but – importantly – is also responsive to the value (to RGCS participants) of knowing about their carrier status for the purposes of reproductive decision-making. Our second argument is that there are normative benefits to deploying utility as an alternative to severity as a criterion for setting the scope of RGCS. In particular, the judgement required to assess the value of information (about carrier status for a genetic condition) for a specific purpose (reproductive decision-making) is preferable to making a value judgement about the lives of people with specific genetic conditions.

We begin by providing an overview of the difficulties in applying severity as an inclusion criterion for RGCS. Next, we introduce the concept of utility and interrogate the relationship between the concepts of severity and utility. Finally, we present our arguments for why we consider it is preferable to focus on utility rather than severity in the context of genomic screening such as RGCS.

## About severity

The terms ‘severity’ and ‘seriousness’ do not have recognised or consensus definitions when applied to diseases in general. This issue was recently raised by the Dutch Health Council when discussing carrier screening:

‘Preconception carrier screening focuses on detecting carrier status of serious hereditary disorders. There is no uniform definition of the concept of severity in hereditary disorders. Whether a hereditary condition can be called serious depends, among other things, on the experience of patients and parents, the experience of healthcare providers and the treatment options [12]. The committee indicates a hereditary condition as serious – and therefore as an important health problem – if the condition causes serious suffering for the future child and/or the future parents’ [13].

The terms ‘severe’ and ‘serious’ are often used interchangeably (as in the above quote), but they can have slightly different connotations. ‘Severe’ is often used in medical terminology to indicate the degree or importance of clinical features, as distinguished from ‘mild’ or ‘moderate’ features. Use of the term ‘severe’ suggests that the condition and its manifestations are at the severe end of a spectrum. ‘Serious’ is not necessarily a measure of the degree or importance of specific clinical features. Rather, ‘serious’ suggests that the consequences of the health condition have a significant impact on the person’s overall health and well-being. Quality of life will vary based on the lived experience of the individual and will be affected by social and environmental conditions. The complexity inherent in the concept of severity means it will be intractably contested and will continue to cause frustration when applied in various decision-making contexts [12, 14]. Due to the influence of personal experience on perceptions of severity, determinations of severity will always have a subjective component.

Often, severe conditions have serious consequences: they can be life-limiting, life-threatening, or have a debilitating impact on quality of life. Life expectancy and mean survival are objective measures of the severity or seriousness of a condition in general, but do not necessarily apply to specific individuals. For example, if the mean life expectancy for a condition is 20 years, specific individuals with this disease could live much longer or shorter lives depending on their access and response to treatment, presence of comorbidities, and a range of other factors. Quality of life assessments are subjective, despite the best efforts to measure QALYs for specific conditions. An additional layer of complexity in the reproductive context is that parents might experience the severity of their child’s condition differently to the child themselves. These variations highlight the tension between severity as a property of a health condition in a general sense and severity as a property of a particular instance of that condition (in a specific person).

The lack of consensus about the definition of severity stems largely from the diversity of lived experience and the subjectivity inherent in assessing quality of life. The multidimensional nature of severity makes consensus about classifying conditions as ‘severe’ or ‘not severe’ very hard to achieve. Attempts have been made to classify conditions according to severity, but these attempts are limited and fraught with challenges [10, 15]. The threshold for severity might vary based on context: policymakers could consider specific conditions as severe when it comes to decision-making about access to treatment for affected individuals, but not severe when it comes to setting the scope of RGCS. In the clinical setting, it can be helpful to keep the definition of severity open or vague, so that individuals or families can decide for themselves if they consider a condition severe enough to warrant certain reproductive choices – based on whether they feel they can or cannot cope with having a child with that specific condition.

In the context of such complexity, determination of severity to guide individual care should ideally happen in a space between individuals/families and clinicians. In the clinical setting, the clinician seeks the best outcome for their specific patient. A shared decision-making process can incorporate both the objective features of the condition and its perceived severity for that patient–family and their specific values and circumstances. However, for purposes of developing a public offer of RGCS a more generalised determination of the severity of candidate conditions is required. Such a determination must precede the decision-making by individuals, their families, and their clinicians. By definition, screening programmes are generally implemented as a ‘one size fits all’ programme that is intended to benefit most people, on balance, and leaves little space for individualisation, and in this way, RGCS is distinct from individual clinical care [16].

When decisions are made about including candidate conditions in an RGCS offer, there is a risk that labelling specific conditions as ‘severe’ could be instrumentalised. In some cases, appeals to the severity of a condition could be used to counter accusations of eugenics, but in other situations, a determination of severity could promote unfavourable value judgements about the lives of people who live with that condition. Such attitudes, particularly when implicit in government-supported health programmes, can contribute to discriminatory societal attitudes towards people who live with genetic conditions and other disabilities [17].

Therefore, determinations of severity about specific conditions, which are necessary if using severity as an inclusion criterion for RGCS, can have unfavourable consequences beyond the RGCS offer itself. We, therefore, suggest that utility might circumvent some of the ethical complexity of using severity – a highly personalised measure of disease impact – for determining the appropriate scope of RGCS.

## About utility

The concept of utility could be considered a more appropriate alternative criterion for inclusion of conditions in RGCS programmes. Broadly, utility refers to fitness for some purpose. It is action-oriented and signifies usefulness: a test for a genetic condition has value to the extent that the information can be used for a specific purpose. The primary purpose of RGCS is ‘to identify couples who have an increased risk of having an affected child in order to facilitate informed decision-making’ [6]. RGCS is aimed at promoting reproductive autonomy, offering reproductive couples the opportunity to make informed decisions about whether and how they wish to conceive by providing information that may be relevant to that decision-making process [2]. There may be other perspectives on the aims of RGCS, namely, that it can be used to reduce disease burden in at-risk populations by helping prevent the birth of children with genetic conditions [18]. However, it is increasingly accepted that promoting reproductive autonomy is the primary aim of RGCS, particularly when offered at scale and supported by public health care systems [16].

Literature on the evaluation of genetic and genomic testing technologies distinguishes two types of utility: clinical utility and personal utility. The clinical utility of a genetic test is the likelihood that its results will affect clinical management and lead to improved health outcomes [19]. A test has clinical utility if it – and any subsequent interventions – leads to improved health outcomes among people with positive test results that outweigh the risks occurring as a result of testing [20]. The clinical utility of a genetic test is affected by a variety of factors including its analytical and clinical validity. These ensure that ‘the relationship between genotype and disease severity is well understood,’ and that there are preventive measures and/or treatment options available [21, 22]. A test has clinical utility if its findings may be relevant for medical management, with available options that are sufficiently safe and effective in bringing about positive outcomes and that are also acceptable, accessible, and affordable.

The personal utility of a genetic test refers to benefits it can have beyond clinical utility [23], when test results ‘can reasonably be used for decisions, actions, or self-understanding which are personal in nature’ [24]. While a broadly shared definition of personal utility is lacking, the literature on personal utility covers a range of personal outcomes including those that are effective (e.g. to enhance coping), cognitive (e.g. value of information) and behavioural (e.g. ability to plan for the future), as well as social outcomes (e.g. research altruism) [25]. Some consider ‘outcomes’ such as satisfying curiosity or addressing privacy concerns as forms of personal utility, too [25]. For the purposes of delineating the scope of RGCS programmes in public health care systems, however, such wide definitions of personal utility are unfit. Tests for carrier status of genetic conditions should not be included in an RGCS offer simply because couples may be curious or have a preference to receive the results. Such an approach is problematic for reasons of equity and potential harms arising from findings that are complex or ambiguous [26]. Rather, conditions should be included in RGCS when they can be used in reproductive decision-making.

For the purposes of this paper, therefore, we are defining personal utility more narrowly and aligned with clinical utility, as the capacity for an RGCS result to influence consideration of reproductive interventions in the context of reproductive decision-making. A test has personal utility when it allows couples ‘to make different (reasonable) choices based on the result. Furthermore, these choices should have the potential to effect positive change in people’s lives’ [24]. This conception of utility links RGCS directly with its primary goal, which is to provide information that could be useful for couples in their reproductive decision-making.

RGCS has both personal and clinical utility. It provides information that couples may use to reconsider having (genetically related) children, or to seek the help of health care professionals in gaining access to preconception reproductive options such as pre-implantation genetic testing (PGT) or prenatal diagnosis, with or without termination of pregnancy. These are deeply personal choices regarding reproduction. Also, RGCS can affect the clinical management of couples who receive an ‘increased chance’ result, as they could become eligible for PGT or prenatal diagnosis.

The use of utility as a selection criterion for establishing the scope of RGCS means that the only conditions that would qualify for inclusion are those for which it is considered worth (re)considering reproductive options. It also means that the utility of tests for genetic conditions included in an RGCS offer will depend on the availability of reproductive options within the health care system where RGCS is offered. These could include various pre-pregnancy or prenatal interventions such as PGT, prenatal diagnosis and termination of pregnancy, and would also depend on the accessibility and cost of such interventions. In order for RGCS to have clinical utility, various reproductive options should be available for the conditions included in RGCS and accessible to the couples eligible for RGCS. Post-test options are an important consideration for jurisdictions that are implementing (or considering) population-wide RGCS.

## Relationship between severity and utility

A shift in emphasis from severity to utility leads to the question of how the usefulness (or utility) of information about carrier status can be determined in the context of reproductive decision-making. Whether taking up various reproductive options is warranted will depend significantly on the severity of the condition in question. Many couples will be willing to consider taking on the risks and burdens associated with PGT or prenatal diagnosis – possibly including, in the case of the latter, termination of a wanted pregnancy – only when it allows them to avoid suffering that will arise from having a child with a severe health condition. Severity, therefore, contributes to utility in a proportional way: the more severe the condition, the more likely it is that information about carrier status for that condition will be considered useful. Tests included in an offer of RGCS are useful when they provide information about conditions that are ‘sufficiently severe’ that taking steps to avoid the condition would be considered acceptable by many prospective parents.

The relationship of severity to utility is also apparent from the selection criteria used in Mackenzie’s Mission. As mentioned earlier, among the criteria are that conditions should be such that an ‘average couple’ would be likely to ‘take steps to avoid the birth of a child with that condition’ [8]. Similarly, an ACMG position statement on RGCS posited that conditions included ‘should be of a nature that most at-risk patients and their partners identified in the screening programme would consider having a prenatal diagnosis to facilitate making decisions surrounding reproduction’ [27]. Both these descriptions relate to the utility of knowing about carrier status for a condition. The concept of utility, therefore, does not do away with considerations of severity. However, while the concept of severity is problematic for reasons outlined above, focusing on utility circumvents some of these problems by placing emphasis on the context in which RGCS is offered, on its aim (i.e. to facilitate reproductive decision-making), and on the value of the information that it can be used to obtain.

## Utility as an inclusion criterion for RGCS – why is it preferable to severity?

In developing an offer of RGCS, decisions need to be made about what conditions to include and exclude from the offer. We suggest that the concept of utility – incorporating clinical and personal utility – is a more appropriate inclusion criterion for RGCS than severity that would function as a necessary but not sufficient criterion. There are two main arguments for preferring to focus on utility rather than severity to set the scope of RGCS.

First, the concept of utility is responsive to the severity of genetic conditions under consideration, but it also makes reference to the goal of carrier screening, namely, to provide information to help prospective parents with reproductive decision-making. So, utility as a criterion for inclusion in RGCS does not discard severity as a consideration, because severity contributes significantly to utility, but considers it in the context of RGCS. As outlined above, severity can be understood as either a property of a health condition or as a property of an instance of that condition in a particular person. Either way, the focus is on the characteristics of (the lives of) people affected by the condition. In the case of RGCS, severity is a concept that is relevant to the potential future child, since it describes a genetic condition that they may have. By contrast, utility is a property of the information obtained via RGCS. Utility, therefore, is relevant to the prospective parents, who are seeking information to support their reproductive decision-making. The severity of the condition that the information is about is an important contributor to the utility of the information, in a scalar way, but utility incorporates additional aspects beyond severity. It is highly responsive both to (individuals’ and families’) context and to the objective of RGCS. Focusing on utility rather than severity shifts the emphasis to the value of the information provided to prospective parents by RGCS for purposes of reproductive decision-making. This shift of emphasis aligns with the most ethically defensible goal of RGCS, namely, supporting reproductive autonomy. Therefore, utility is a more relevant criterion that assesses prospective parents’ potential to benefit from RGCS.

Focusing on the utility of the information provides a mechanism for considering the context of reproductive decisions, including the options available to the couple seeking RGCS. It provides a route for ensuring RGCS coheres with other available interventions, such as newborn screening and available treatments for genetic conditions. Determinations of utility vary according to the condition’s severity, but also factor in how useful knowing about carrier status for that condition will be for clinical management and reproductive decision-making. This approach also allows that information obtained via RGCS can have both personal utility (to inform personal reproductive choices) and clinical utility. Thus, as a criterion for setting the scope of RGCS, utility is more appropriate as it is clearly linked to the context and purpose of the test.

Second, there are ethical reasons for emphasising the concept of utility rather than severity in the context of inclusion criteria for RGCS. As mentioned, utility is a property of the information gleaned via RGCS, while severity is a property of a genetic condition. Determinations of severity imply value judgements about the relative quality of the lives of people who live with that condition, and potentially also about the life of a future child. It is ethically problematic, particularly for governments or medical professionals, to make a judgement about the value of an individual’s life, or the collective value of the lives of people who live with a specific genetic condition [11, 28]. Yet at a policy and programmatic level, decisions need to be made about which conditions to include in an offer of RGCS. However, assessing the utility of knowing about carrier status for a genetic condition places severity in the context of RGCS and is, therefore, more acceptable, even if it does require some assessment of severity. The focus on utility connects those policy-level decisions more closely to the primary goal of RGCS, which is to provide individuals and families with information that is useful for reproductive decision-making.

## Conclusion

Decisions about which conditions and genes to include in RGCS require careful consideration. We have argued that the criterion of severity could be replaced by utility, for two main reasons. First, while severity of the genetic condition identified is among the contributors to the utility of an RGCS result, utility is a concept that is more responsive to the context and purpose of screening than severity. While severity is contextual in the sense that people’s experiences and context affect their perception of severity, utility of an RGCS result is also determined by features of the health care system in which the screening is offered – notably the availability of reproductive options – and the goal of RGCS, namely to facilitate reproductive decision-making. Second, we consider utility to be ethically preferable because it is a property of a genetic test result (information about carrier status), as opposed to severity which is either a property of a genetic condition in a general sense, or a property of an instance of a genetic condition (in a specific person). Determinations of severity require value judgements about the lives of people with genetic conditions, while determinations of the utility of a screening result are about the value of that information in relation to a specific purpose (i.e. reproductive decision-making). To deploy the concept of utility as an inclusion criterion for RGCS – or other forms of genetic screening or testing – would require a more comprehensive analysis than we can provide here, but we have offered some initial suggestions for the clinical and personal dimensions of utility that begin to set out the considerations for a concept of utility that could be used to determine the appropriate scope of population-wide RGCS programmes.
